# The influence of parents’ education anxiety on children’s learning anxiety: the mediating role of parenting style and the moderating effect of extracurricular tutoring

**DOI:** 10.3389/fpsyg.2024.1380363

**Published:** 2024-06-05

**Authors:** Xia Yin, HePing Zhang, Meng Chen

**Affiliations:** ^1^Institute of Educational Sciences, Hubei University of Education, Wuhan, China; ^2^School of Education, Huazhong University of Science and Technology, Wuhan, China; ^3^School of Economics and Management, Nanchang Institute of Science and Technology, Nanchang, China; ^4^Normal School of Vocational Techniques, Hubei University of Technology, Wuhan, China

**Keywords:** educational anxiety, learning anxiety, parenting style, extracurricular tutoring, academic competition

## Abstract

**Introduction:**

This study investigates the intricate relationship between parents’ education anxiety and children’s learning anxiety, examining the mediating role of parenting style and the moderating effect of extracurricular tutoring.

**Methods:**

Utilizing data from the “Survey of Parents and Students in Primary and Secondary Schools,” the study employs stratified sampling (*n* = 3,298) and various psychological scales to measure education anxiety, parenting styles, and extracurricular tutoring.

**Results:**

This study reveals that parents’ education anxiety significantly influences children’s learning anxiety, with a notable positive correlation (*r* = 0.301^**^). Parenting styles particularly rejection and overprotection style increase this anxiety, while emotional warmth style decreases it. Academic tutoring serves as a moderator, reducing the impact of parental anxiety on children’s learning anxiety (*β* = −0.033, *p* < 0.05).

**Discussion:**

The study underscores the importance of addressing internal family dynamics to alleviate education anxiety. It advocates for a balanced approach to tutoring, emphasizing the benefits of arts and sports activities in reducing learning anxiety. Parents should be encouraged to adopt emotionally warm parenting styles and to engage their children in a variety of extracurricular activities.

## Introduction

In recent years, education anxiety has emerged as a pervasive social phenomenon, particularly manifesting in the psychological experiences of parents in primary and secondary school settings. Current research primarily focuses on unraveling the societal underpinnings of this issue. Externally, this includes the dearth of accessible, high-quality educational resources (Yang, 2019), the evaluative and selection practices within education systems (Qian and Miao, 2021), the dependence on supplementary tutoring (Ma and Liu, 2021), and the extensive impact of the mass media (Geng, 2021). Internally, factors such as heightened parental expectations (Yin et al., 2022), tendencies toward social comparison and conformity (Zeng, 2020), and the role of family socio-economic status (Gong et al., 2021) are also significant. However, this extensive body of research often neglects a crucial aspect: the effect of parents’ education anxiety on children’s comprehensive development. As an integral element of the social fabric, this anxiety does not operate in isolation but profoundly affects children. The present scenario among primary and secondary school students witnesses intense academic competition, as evidenced by the Program for International Student Assessment (PISA) 2018 data, which indicates a global average of 44 h per week spent on studying. Research has highlighted that 70.03% of students experience learning anxiety (Ding, 2023), with test anxiety affecting 22.32% of students (Hou et al., 2023). Furthermore, there is a rise in severe psychological issues such as fear of learning, anxiety disorders, and depression among students. Some studies have started to recognize the detrimental impacts of parents’ anxiety on children’s development, including limitations on academic performance (Gao et al., 2023; Tian and Wang, 2024), as well as increases in emotional and externalizing behavior problems (Bayer et al., 2006). However, limited attention has been given to the intergenerational transmission of anxiety between parents and their offspring.

In the shadow of parents’ education anxiety, can children grow freely under the anxious gaze of their parents? Existing research highlights that education anxiety readily triggers behaviors associated with “hothousing” and overparenting. This manifests in two main ways: first, as a robust increase in family education expenditures. Data from the China Education Panel Survey (CEPS) reveal a year-on-year rise in such spending, with 2018 expenditure for extracurricular tutoring being 3.86 times that of 2010 (Xue and Zuo, 2021), and the average number of extracurricular activities per primary and secondary school student reaching 2.54 (Zhang et al., 2021); Second, it manifests as increasingly meticulous parenting practices. The prevalence of high-engagement, high-intensity, and controlling “helicopter parenting” styles is on the rise, with parents acting as their children’s “educational brokers” (Yang, 2018). However, the academic returns from such extracurricular tutoring come at the cost of sacrificing children’s psychological well-being (Zhang and Gao, 2022), resulting in the emergence of sensitive, fragile, and anxious “orchid children” (Ellis and Boyce, 2018). Past research began to construct an understanding of how parents’ education anxiety, by increasing academic burdens and altering parenting behaviors, impacts the development of their children. Within the framework of emotional contagion theory, it is posited that emotions within a family are synchronized or mutually influential. Therefore, it is inferred that parents’ education anxiety might be transmitted to children through tutoring and parenting styles, thereby inciting education anxiety in children.

The practical significance of this study lies in forging a path for the internal governance of education anxiety within families. Currently, measures to alleviate education anxiety primarily focus on improving the external environment, such as increasing high-quality educational resources, reforming the educational evaluation system, and combating news media that exaggerate and sensationalize anxiety. However, these measures have not effectively mitigated parents’ education anxiety, in part due to a lack of consideration for internal family factors. The most crucial aspect of internal governance is to prompt parental reflection and awakening regarding education. If parents realize that their own anxiety can be transmitted to their children, causing the children to experience learning anxiety and subsequently affecting their academic and psychological development, they will consciously control their anxiety and actively improve their family education capabilities. In summary, to address education anxiety from a micro-family perspective, it is necessary to explore the mechanism of impact between parents’ education anxiety and children’s learning anxiety. Based on this, our study attempts to utilize data from the “Survey of Parents and Students in Primary and Secondary Schools” to analyze the influencing factors of family education anxiety and its quantitative relationship with children’s learning anxiety, in hopes of finding effective strategies to alleviate education anxiety.

## Theoretical framework and hypotheses

The emotional states and behavioral attitudes of individuals or groups can synchronously affect both parties in social interactions. The theory of emotional contagion posits that emotional contagion is an unconscious transmission process of emotional experiences between individuals (Hatfield et al., 1994). This mechanism generally occurs at the following four levels: (1) The individual-level mimicry-feedback mechanism. Hatfield et al. (1993) argue that individuals, upon perceiving others’ facial expressions, voices, postures, and movements, engage in mimicry, which, through corresponding feedback and stimuli, blends with the emotional state of the other (Hatfield et al., 1993). (2) The inter-individual level contagion effect mechanism. This level primarily reflects the variability in influence efficacy among individuals. Researchers have found that emotional contagion varies depending on individuals’ gender, profession, age, and personality traits (Weisbuch and Ambady, 2008). (3) The interpersonal interaction level emotional transmission mechanism. In interpersonal communication, shared emotions can affect behavioral outcomes, such as customers being influenced by the negative emotions of service staff (Barsade, 2002), or leaders’ positive emotions impacting employees’ attitudes, behaviors, and performance (Tee, 2015). (4) The group level emotion formation and transmission mechanism. The emotions of team members constructively contribute to group-level emotions, with higher member familiarity strengthening group emotions (Hatfield et al., 2014). In this study, the emotional contagion between parents and children is explored from the third level, examining the emotional contagion mechanism in parent–child interactions, and from the family group level, presenting the results of emotional transmission.

Building upon the fourth level of the theory of emotional contagion, emotions within a group are interconnected over time, gradually becoming synchronized or interactive, with the contagion being stronger among more familiar team members (Hatfield et al., 1994; Barsade, 2002). A family, as a relatively closed and tightly-knit organizational unit characterized mainly by affection and companionship, fosters an environment where emotions are more contagious among its members. Based on this understanding, the following research hypothesis is proposed: Parents’ education anxiety positively influences the level of children’s learning anxiety (*H1*).

Based on the third level of the theory of emotional contagion, individuals in interpersonal interactions are consciously or unconsciously induced by the emotions of other members to share emotions, thereby affecting behavioral outcomes. Tee (2015) divides emotional contagion into implicit and explicit emotional contagion, with the former being an unconscious process and the latter a conscious one. Parents’ education anxiety implicitly affects parenting styles, manifested in changes in educational beliefs and attitudes toward children when nurturing them. Existing research widely acknowledges that negative parenting styles are likely to lead to psychosomatic issues in children, such as more depression and anxiety under controlling parenting (Bruggen et al., 2008). Parents with high anxiety, fearing that the external environment might threaten their children’s development, tend to adopt overprotective and controlling parenting styles out of a desire to protect their children (“for their own good”), which can exacerbate children’s anxiety levels. In contrast, emotionally warm parenting styles can help reduce students’ anxiety levels (Liu, 2014; Xue, 2016; Sun and Tang, 2019). Based on these findings, this study infers that parents’ education anxiety affects their own parenting styles, which in turn influences children’s learning anxiety. Thus, the following research hypothesis posited: Parenting style plays a mediating role between their education anxiety and children’s learning anxiety (*H2*).

Explicit emotional contagion in the context of parents’ education anxiety manifests in direct behavioral interventions in children’s learning, the most common of which is consciously increasing extracurricular tutoring for the child. Research has shown that overly anxious families tend to focus solely on “academic” tutoring, while families with normal levels of anxiety pay attention to both “academic” and “arts and sports” types of tutoring for their children (Gong et al., 2021). Different types of tutoring have varied impacts on children’s academic and psychological well-being. Current research on academic tutoring predominantly supports its positive role in enhancing academic performance (Xue, 2016; Xue and Song, 2018), and there’s an observed “placebo effect” on academic emotions (Liu, 2014; Sun and Tang, 2019). Consequently, a greater amount of academic tutoring might transform parental education anxiety, offsetting its negative impact on students’ learning anxiety. In contrast, less academic tutoring might amplify the positive influence of parental education anxiety on student learning anxiety. Based on this understanding, the research hypothesis is proposed: The impact of parents’ education anxiety on students’ learning anxiety is moderated by the amount of academic tutoring (*H3a*).

**Figure 1:**
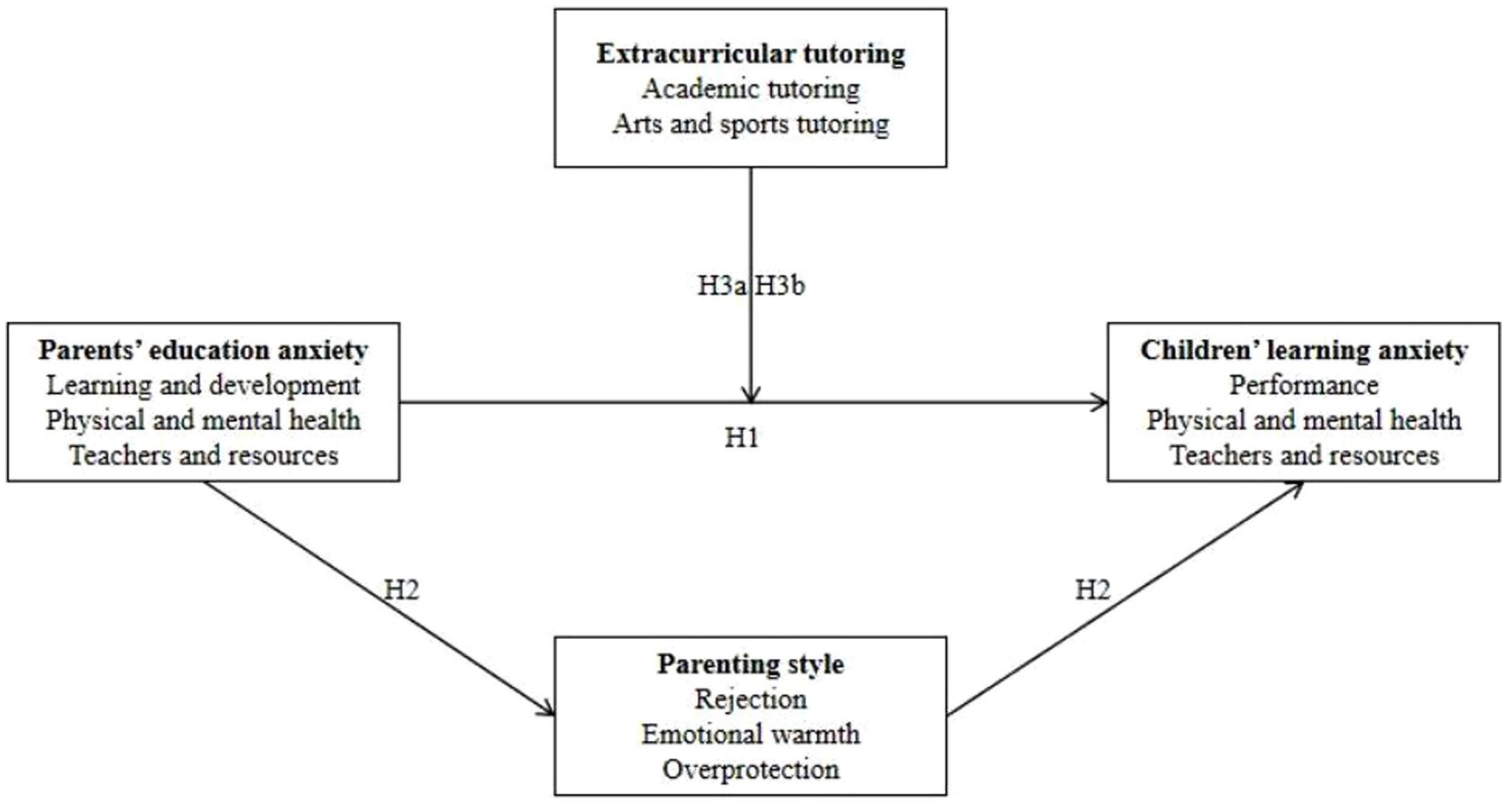
Conceptual framework.

Previous research supports a negative correlation between tutoring in the arts and sports and parents’ education anxiety (Zhang et al., 2021), indicating that parents who emphasize their children’s tutoring in these areas tend to exhibit lower levels of education anxiety. Currently, there is scant evidence supporting the impact of arts and sports tutoring on academic performance and emotional states related to schooling. Based on this observation, research hypothesis is proposed: Arts and sports tutoring does not serve as a moderating factor in the relationship between parents’ education anxiety and children’s learning anxiety (*H3b*) (Figure 1).

## Materials and methods

### Participants

The data for this study were sourced from a survey conducted by the Hubei Teacher Education Research Center and the Student Development Collaborative Research Center, targeting parents and students in primary and secondary schools. The survey employed stratified sampling, selecting 18 primary and secondary schools from five provinces—Hubei, Zhejiang, Guangdong, Jiangsu, and Hunan—based on convenience. Within these selected schools, classes were sampled, and surveys were conducted among all students and parents of the sampled classes. During the implementation process, head teachers of the sampled classes first organized students and parents with unique corresponding codes. Then, students and parents filled out the questionnaires based on their unique identifiers. Finally, the codes of students and parents were effectively linked to form a matched set of parent-student data. The survey collected 7,419 student questionnaires and 6,703 parent questionnaires. After data cleaning, which involved removing unmatched parent questionnaires and samples with non-corresponding invitation codes, a total of 3,298 complete and valid matched parent-student samples were obtained. Among these, 498 parents (15.1%) had received tertiary education or above, 1,040 (31.5%) had completed high school or vocational tutoring, and 1,760 (53.4%) had education levels of middle school or below; 1,431 students (43.4%) were in primary school, 1,396 (42.3%) were in middle school, and 471 (14.3%) were in high school.

### Measures

#### Measurement of parents’ education anxiety

In this study, education anxiety is distinctly different from trait anxiety. Education anxiety is essentially a state anxiety, characterized by emotions such as tension, worry, and panic that parents experience during the process of educating their children (Chen and Xiao, 2014). It fluctuates with changes in children’s academic performance or other educational factors and may decrease or even disappear once the child leaves the educational setting. In contrast, trait anxiety is more of a personality trait, characterized by its situational breadth and greater stability. The measurement of parents’ education anxiety in this research utilized the Parental Educational Anxiety Scale developed by Yin et al. (2022). This scale includes three dimensions: anxiety about learning and development, anxiety about physical and mental health, and anxiety about teachers and resources. Anxiety about learning and development refers to parents’ concerns about their children’s current academic performance and future prospects, such as concerns about children’s grades, getting into a good university, or finding a good job; anxiety about physical and mental health involves concerns about the children’s physical (exercise, sleep, personal safety, etc.) and psychological aspects (personality, interpersonal relationships, etc.), such as worries about bullying at school, the nutritional safety of school meals, or whether the child has friends; anxiety about teachers and resources pertains to parents’ concerns about the quality of school education, the availability of quality educational resources, such as whether the school is a key school, whether the class teacher is a renowned educator, and the reputation of tutoring institutions.

To verify the reliability and validity of the questionnaire, the data sample was split into two halves. The first half of the valid samples (*n* = 3,351) was used for the Kaiser-Meyer-Olkin (KMO) test and Bartlett’s test of sphericity. The results showed a KMO value of 0.885 and a Bartlett’s test value of 14888.36 (*p* < 0.001), indicating that the sample data was suitable for factor analysis. Subsequently, Principal Component Analysis (PCA) with varimax rotation was used to extract the number of factors, identifying three factors with eigenvalues greater than 1, accounting for a cumulative total variance of 62.24%. The second half of the sample (*n* = 3,352) was then used to test the validity of the 12 items identified. The results of the Education Anxiety Scale showed a χ^2^/df ratio of 3.484, Comparative Fit Index (CFI) of 0.953, Goodness of Fit Index (GFI) of 0.905, Tucker-Lewis Index (TLI) of 0.952, Root Mean Square Error of Approximation (RMSEA) of 0.071, and Root Mean Square Residual (RMR) of 0.032. All these fit indices reached an acceptable range, indicating good model fit. In this study, the internal consistency coefficient (Cronbach’s alpha) for the scale was 0.874, demonstrating that the scale has good reliability and validity. These findings corroborate the utility of the scale as a reliable and valid instrument for gaging parents’ education anxiety.

#### Measurement of learning anxiety

Learning anxiety refers to the negative emotional states of tension, worry, and fear experienced by students during learning activities (Collie et al., 2017). Some researchers have narrowed this concept to focus on subject-specific anxieties, like anxiety related to foreign languages (Horwitz et al., 1986) or mathematics (Richardson and Suinn, 1972), or specifically exam anxiety (Sarason et al., 1978). In this study, learning anxiety is conceptualized as a pervasive form that can occur at any time, place, or in relation to any aspect of learning. Consistent with the dimensions of parents’ education anxiety, students’ learning anxiety was categorized into three dimensions: performance anxiety, physical and mental health anxiety, and teachers and resources anxiety. This classification took into consideration the intrinsic characteristics of students. The data of 7,419 students was similarly split into two halves for analysis. The results of the first half of the data indicated a KMO measure of 0.927 and a Bartlett’s test value of 21387.05 (*p* < 0.001), demonstrating the suitability of the data for factor analysis. Three common factors explained a cumulative variance of 61.17%. The Confirmatory Factor Analysis (CFA) of the second half showed a χ^2^/df ratio of 4.776, CFI of 0.898, GFI of 0.911, TLI of 0.952, RMSEA of 0.085, and RMR of 0.042. These fit indices reached acceptable levels, indicating a good model fit. Cronbach’s alpha for this learning anxiety scale was 0.913, suggesting that the scale has good reliability.

#### Measurement of parenting styles

This study utilized the Shortened Egna Minnen Betraffende Upfostran-Chinese (S-EMBU-C), revised by Jiang et al. (2010). The S-EMBU-C consists of separate versions for fathers and mothers, each containing 21 items. The questionnaire is divided into three dimensions: rejection, emotional warmth, and overprotection. It employs a 4-point Likert scale for scoring (1-never, 2-seldom, 3-often, 4-always), with higher scores indicating higher levels of parental rejection, overprotection or emotional warmth. In line with previous research, this study combined the dimensions of fathers and mothers into a single parental dimension to measure the overall family parenting style. The Cronbach’s alpha of the scale in this study was 0.794, indicating a good level of reliability. This approach reflects an integrated assessment of the family’s parenting style, providing insights into the collective influence of both parents on their children.

#### Measurement of extracurricular tutoring

The study addressed the measurement of extracurricular tutoring, categorizing it into academic tutoring and arts/sports tutoring. Specific survey items were employed to gage each type. For academic tutoring, participants were queried about the number of academic subject tutoring classes (such as language, mathematics, foreign languages, biology, chemistry, etc.) attended by their child in the past year. Similarly, for arts and sports tutoring, inquiries were made regarding the attendance of arts and sports classes (such as music, chess, calligraphy, painting, sports, etc.).

This study employs SPSS 25.0 for descriptive and correlation analyses; uses Amos for confirmatory structural analysis and mediation effect testing on various scales; and utilizes the PROCESS plugin for testing moderation effects.

## Results

### Common method bias test

To address potential common method bias, this study employed several control strategies during the survey process. These included the implementation of anonymous surveys and the incorporation of reverse-scored items. Moreover, Harman’s single-factor test was utilized to assess the presence of common method bias. The analysis yielded 17 factors with eigenvalues exceeding 1, with the first factor accounting for 14.47% of the variance. This percentage falls well below the critical threshold of 40%, thereby suggesting that common method bias is not a significant concern in this dataset.

### Descriptive and correlative analysis

The results presented in Table 1 reveal a notable positive correlation between parents’ education anxiety and children’s learning anxiety (*r* = 0.301^**^), indicating that higher levels of parents’ education anxiety correlate with increased learning anxiety among children. Additionally, there is a discernible correlation between parents’ education anxiety and their parenting styles. Specifically, parents’ education anxiety is positively correlated with overprotective and rejecting parenting styles (*r* = 0.166^**^, *r* = 0.159^**^), and negatively correlated with emotional warmth (*r* = −0.133^**^). Parenting styles also show significant correlations with children’s learning anxiety, where overprotective and rejecting styles positively correlate with learning anxiety (*r* = 0.223^**^, *r* = 0.219^**^), and emotional warmth negatively correlates (*r* = −0.204^**^).

**Table 1:** Descriptive statistics of the variables and the correlations between variables.

	*M*	SD	1	2	3	4	5	6	7
Education anxiety	3.05	0.78	–						
learning anxiety	2.53	0.84	0.301**^a^	–					
Rejection	1.55	0.56	0.166^**^	0.223^**^	–				
Emotional warmth	3.06	0.72	−0.133^**^	−0.204^**^	−0.293^**^	–			
Overprotection	2.18	0.51	0.159^**^	0.219^**^	0.577^**^	−0.071	–		
Arts/sports tutoring	2.33	1.40	−0.065^**^	−0.083^**^	−0.016	0.096^**^	−0.015	–	
Academic tutoring	1.63	1.08	0.027	0.005	0.041^*^	0.046^**^	0.058^**^	0.280^**^	–

a **p < 0.01.

### Mediating effects of parenting styles on parents’ education anxiety and children’s learning anxiety

**Figure 2:**
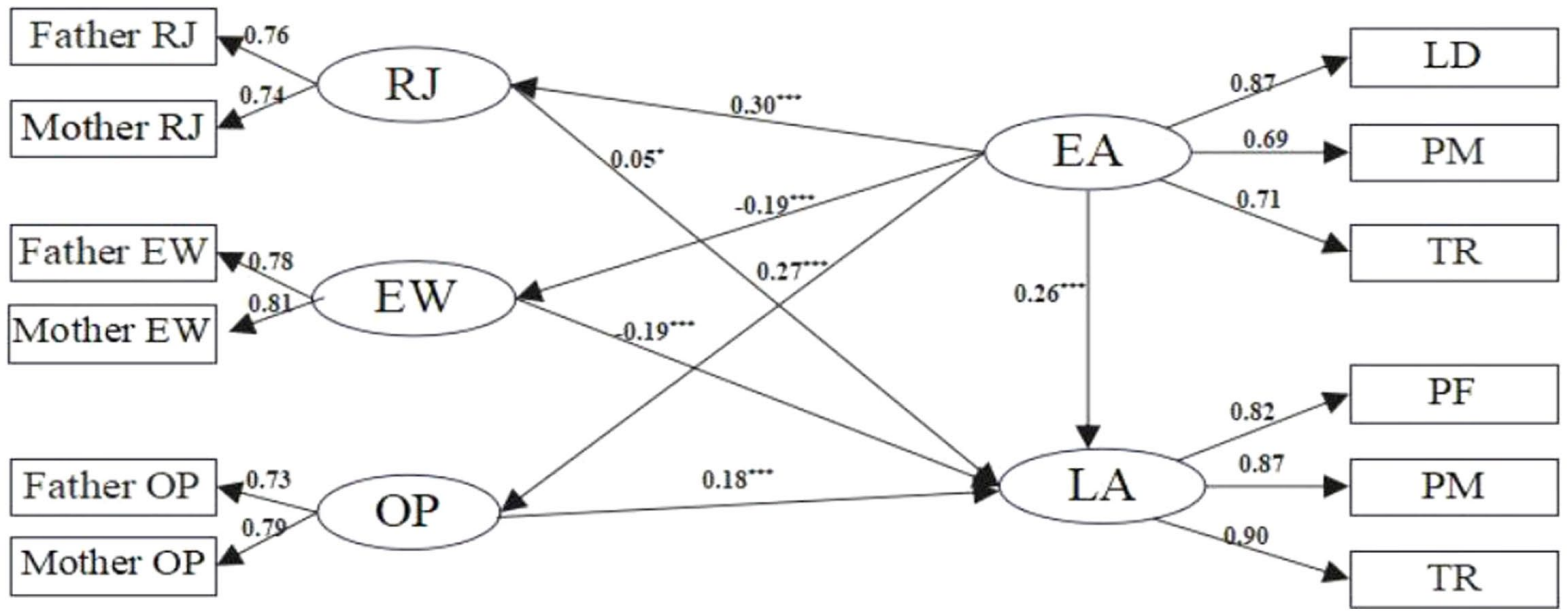
The mediation model of parenting style. RJ, rejection; EW, emotional warmth; OP, overprotection; EA, education anxiety; LA, learning anxiety; LD, learning and development; PM, physical and mental; TR, teachers and resources; PF, performance.

The identified significant correlations among parents’ education anxiety, parenting styles, and children’s learning anxiety provided a basis for mediation effect testing. Employing a structural equation model (SEM), the study achieved favorable fit indices (χ^2^/df = 4.411, RMR = 0.022, GFI = 0.995, AGFI = 0.990, NFI = 0.987, RFI = 0.979). As depicted in Figure 2, parents’ education anxiety was found to positively predict children’s learning anxiety (*β* = 0.26, *p* < 0.001), signifying that heightened levels of parents’ education anxiety contribute to increased learning anxiety in children. When integrating parenting styles into the model, it was observed that parental education anxiety influences children’s learning anxiety positively through overprotective and rejecting parenting styles, and negatively through emotion warmth styles.

To further verify the mediating role of parenting styles, this study employed the Bootstrap percentile method (2000 iterations) for testing. If the 95% confidence interval of the indirect effect does not include zero, it indicates a significant mediation effect; otherwise, there is no mediation effect. As shown in Table 2, the confidence intervals of all three mediating paths for the impact of parents’ education anxiety on children’s learning anxiety do not include zero, confirming the mediation effect. In path 1, the mediation effect of education anxiety—rejection—learning anxiety is 0.015, accounting for 15% of the total effect; in path 2, the mediation effect of education anxiety—emotional warmth—learning anxiety is 0.036, accounting for 36%; and in path 3, the mediation effect of education anxiety—overprotection - learning anxiety is 0.049, accounting for 49%.

**Table 2:** Mediation effect test of parenting style.

Path	Standardized effect size	Bootstrap SE	Bootstrap 95% confidence interval	Percentage of mediation effect
Education anxiety—rejection—learning anxiety	0.015	0.001	[0.008–0.024]	15%
Education anxiety—emotional warmth—learning anxiety	0.036	0.021	[0.011–0.048]	36%
Education anxiety—overprotection—learning anxiety	0.049	0.005	[0.032–0.053]	49%

### Moderating effect of extracurricular tutoring on the relationship between parents’ education anxiety and children’s learning anxiety

The moderating role of extracurricular tutoring was explored using PROCESS for moderation analysis. The results, as shown in Table 3, indicate that the interaction term coefficient between education anxiety and academic tutoring is significant (*β* = −0.033, *p* < 0.05), implying that the extent of academic tutoring moderates the relationship between parents’ education anxiety and children’s learning anxiety. Conversely, the interaction term between education anxiety and arts and sports tutoring is not significant (β = −0.029, *p* > 0.05), suggesting that arts and sports tutoring does not moderate this relationship. Interestingly, the data indicates that arts and sports tutoring has a negative impact on children’s learning anxiety (β = −0.058, *p* < 0.01), denoting that increased engagement in arts and sports tutoring is associated with lower levels of learning anxiety.

**Table 3:** Moderating effect analysis of extracurricular tutoring.

Independent variables	Dependent variables: children’s learning anxiety		
	Model 2	Model 3	Model 4
Academic tutoring	0.024	0.026	0.026
Arts/sports tutoring	−0.055**^a^	−0.054**	−0.058**
Education anxiety	0.283***^b^	0.282***	0.283***
Education anxiety*academic tutoring		−0.033*	
Education anxiety*arts/sports tutoring			−0.029
Adjust *R*^2^	0.100	0.104	0.100
F	31.429***	29.347***	29.257***

a **p < 0.01.

b **p < 0.001.

**Figure 3:**
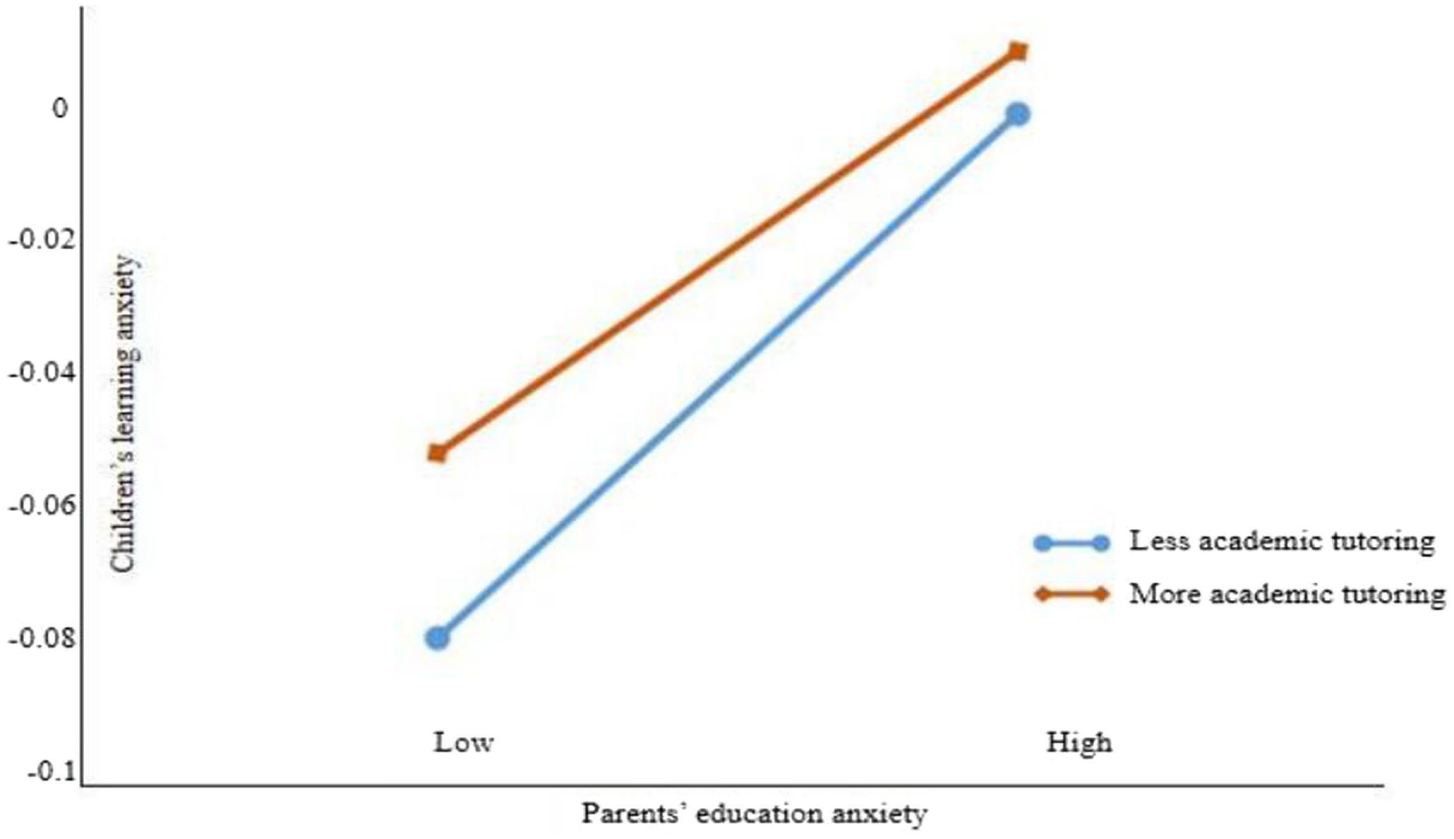
The moderating effect diagram of academic tutoring.

To further clarify the moderating effect of academic tutoring, as shown in the simple slope analysis in Figure 3, when the frequency of academic tutoring sessions is low, parents’ education anxiety has a higher predictive effect on children’s learning anxiety. As the number of academic tutoring sessions increases, the impact of parents’ education anxiety on children’s learning anxiety decreases. Specifically, for every unit increase in the moderator variable, the number of academic tutoring sessions, the impact of parents’ education anxiety on children’s learning anxiety decreases by 0.033 units.

## Discussion

This study initiated an examination of the prevalent phenomenon of education anxiety, focusing on the emotional contagion mechanism through which parents’ education anxiety impacts their children’s learning anxiety within the family environment. This inquiry is especially relevant in light of the growing acknowledgment of the family’s significant influence on the emotional and academic development of children. Initially, we discovered that parents’ education anxiety significantly and positively impacts their children’s learning anxiety. This finding corroborates the hypothesis H1, aligning with the theory of emotional contagion. Grounded in the ecosystem theory, the family is conceptualized as the individual’s microsystem, exerting a profound influence on the individual’s development. This study underscores that when parents exhibit anxiety concerning their children’s education, such anxiety is directly perceived by the children, thereby becoming a contributing factor to their learning anxiety.

Further, the transmission of parents’ education anxiety to their children is significantly mediated by parenting styles. It was found that rejection and overprotection parenting styles exacerbate the impact of parents’ education anxiety on children’s learning anxiety, whereas emotional warmth style has a mitigating effect. This finding, confirming hypothesis H2, echoes the results of Gao et al. (2023), highlighting the mediating role of negative parenting styles in the nexus between education anxiety and adolescent emotional well-being. Anxious parents often adopt an intensive parenting approach, exerting strict control over their children’s academic and personal lives, which can stifle the development of self-belief and lead to anxiety. Research by Yang and Hou (2009) supports this, demonstrating a positive correlation between children’s anxiety and negative parenting styles, such as overprotection and rejection, and a negative correlation with positive styles, such as emotional warmth style. This indicates that children are not only directly affected by their parents’ anxiety but also indirectly through the comprehensive influence of parenting styles on their learning anxiety.

Lastly, the study investigated the moderating role of extracurricular tutoring in the relationship between parents’ education anxiety and children’s learning anxiety, confirming hypothesis H3. Academic tutoring was found to play a negative moderating role, while arts and sports tutoring did not exhibit a moderating effect. An increase in academic tutoring sessions was observed to mitigate the impact of parents’ education anxiety on children’s learning anxiety, albeit with a modest impact coefficient of 0.033. This suggests that academic tutoring may serve as a conduit for transforming and alleviating some of the parents’ education anxiety, thereby lessening its influence on children. This interpretation of these findings may be influenced by the cultural perspectives of our study participants. Given that our data originates from China, where schools predominantly emphasize academic achievement in selecting students, it follows that parents are highly anxious about their children’s grades, and children themselves are equally concerned about academic performance. When parents provide additional subject-specific tutoring for their children, it can mitigate the intensity of the anxiety transmission chain between parents and children. Conversely, arts and sports tutoring directly and significantly reduces students’ learning anxiety, indicating its potential to enhance non-cognitive abilities (Fang, 2018), and suggesting that parents seeking to alleviate their children’s learning anxiety should consider increasing engagement in arts and sports over merely academic tutoring sessions.

## Implications and conclusion

This research unveils the intricate mechanisms influencing family education anxiety, offering substantial theoretical and practical implications. Theoretically, it pioneers a dual-perspective approach, integrating insights from both parents and students. This method diverges from traditional models that solely focus on parents’ perspectives, thus neglecting the agency of the student. By correlating data collected from parents about family education with data from students on their learning experiences, a more holistic view emerges, capturing the diverse perspectives and beliefs of these key stakeholders. This approach objectively illuminates the impact of parent–child relationships on education anxiety. Moreover, the study elucidates the nature of family education anxiety, encompassing concerns about learning development, mental and physical health, and the quality of educational resources. This clarity in definition addresses the previously ambiguous concept of education anxiety. Significantly, the research introduces an integrated theoretical model that links family situational factors, education anxiety, and learning anxiety, unveiling the emotional contagion mechanism within familial settings. This model not only applies the theory of emotional contagion in a new context but also expands its scope. Practically, the identified factors affecting family education anxiety provide essential insights for reforming fundamental education in China and mitigating widespread societal education anxiety. The insights into the interaction between family education anxiety and student learning anxiety are crucial for enhancing parental educational strategies, fostering positive family learning environments, and promoting a scientific approach to parenting. This study suggests strategies to improve the mental and physical well-being of primary and secondary school students, addressing the root causes of parents’ education anxiety and children’s learning anxiety.

For administrators and policymakers in China’s fundamental education system, a broad societal perspective on education anxiety is imperative. Recognizing that education anxiety is a symptom of broader societal issues, mere educational reform might be insufficient (Peng and Dang, 2021). The study highlights the deep societal conflicts reflected in family education anxiety during China’s transitional phase, marked by market economy influences, consumerism, competition, and cultural clashes. Current educational reforms, primarily aimed at altering evaluation methods, reducing academic burdens, and enhancing in-school education quality, need to transcend educational boundaries and integrate with social security systems to establish a balanced educational ecosystem. For parents, understanding the impact of their anxiety on their children’s emotions is crucial. Conscious efforts to control this anxiety can significantly benefit their children’s learning psychology. Emphasizing emotional communication, reducing academic pressure, and avoiding excessive interference in their children’s learning and life are recommended. Overbearing behaviors can harm parent–child relationships and negatively impact children’s mental and physical development. Moderation in academic tutoring and increased emphasis on arts and sports can alleviate student learning anxiety and promote social and emotional skills development.

This research also has certain limitations. Firstly, although the study attempted to cover a broad range of educational stages, urban and rural areas, and diverse characteristics, it was conducted based on convenience sampling in only some provinces and cities, not encompassing the entire country. Future studies should include underdeveloped western regions as well as areas with intense educational resource competition, such as Beijing and Shanghai. Secondly, while the scales used in this study have been tested and found to have good reliability and validity, they are self-reported and thus susceptible to subjective biases. Future research could employ triangulation methods to enhance the validity of the data. Lastly, given the data sample, it is impossible to exhaust all influencing factors. This research is exploratory and empirically based on theoretical foundations. Future studies are needed to further explore causal relationships.

## Data availability statement

The original contributions presented in the study are included in the article/supplementary material, further inquiries can be directed to the corresponding author.

## Ethics statement

The studies involving humans were approved by Ethics Committee of Hubei University of Education. The studies were conducted in accordance with the local legislation and institutional requirements. Written informed consent for participation in this study was provided by the participants’ legal guardians/next of kin.

## Author contributions

XY: Conceptualization, Data curation, Investigation, Writing – original draft. HZ: Funding acquisition, Methodology, Resources, Supervision, Writing – review & editing. MC: Formal analysis, Investigation, Methodology, Resources, Supervision, Writing – review & editing.
